# The genetic landscape of inherited eye disorders in 74 consecutive families from the United Arab Emirates

**DOI:** 10.1002/ajmg.c.31824

**Published:** 2020-08-11

**Authors:** Cécile Méjécase, Igor Kozak, Mariya Moosajee

**Affiliations:** ^1^ Institute of Ophthalmology University College London London UK; ^2^ Moorfields Eye Hospitals United Arab Emirates (UAE) Dubai UAE; ^3^ Moorfields Eye Hospital NHS Foundation Trust London UK; ^4^ Great Ormond Street Hospital for Children NHS Trust London UK; ^5^ The Francis Crick Institute London UK

**Keywords:** founder mutation, genetic testing, next generation sequencing, targeted gene panels, United Arab Emirates

## Abstract

Genetic eye diseases are phenotypically and genetically heterogeneous, affecting 1 in 1,000 people worldwide. This prevalence can increase in populations where endogamy is a social preference, such as in Arab populations. A retrospective consecutive cohort of 91 patients from 74 unrelated families affected with non‐syndromic and syndromic inherited eye disease presenting to the ocular genetics service at Moorfields Eye Hospitals United Arab Emirates (UAE) between 2017 and 2019, underwent clinically accredited genetic testing using targeted gene panels. The mean ± *SD* age of probands was 27.4 ± 16.2 years, and 45% were female (41/91). The UAE has a diverse and dynamic population, and the main ethnicity of families in this cohort was 74% Arab (*n* = 55), 8% Indian (*n* = 6) and 7% Pakistani (*n* = 5). Fifty‐six families (90.3%) were genetically solved, with 69 disease‐causing variants in 40 genes. Fourteen novel variants were detected with large deletions in *CDHR1* and *TTLL5*, a multiexon (1–8) duplication in *TEAD1* and 11 single nucleotides variants in 9 further genes. *ABCA4*‐retinopathy was the most frequent cause accounting for 21% of cases, with the confirmed UAE founder mutation c.5882G>A p.(Gly1961Glu)/c.2570T>C p.(Leu857Pro) in 25%. High diagnostic yield for UAE patients can guide prognosis, family decision‐making, access to clinical trials and approved treatments.

## 1 INTRODUCTION

Inherited eye disease is phenotypically and genetically heterogeneous with over 430 known disease‐causing genes (Patel et al., 2019). Approximately 1 in 1,000 people worldwide are affected with either progressive, non‐progressive, syndromic, or non‐syndromic genetic pathologies falling into the spectrum of development eye disorders, corneal and retinal dystrophies, and/or hereditary optic neuropathies (Stone, 2007). Retinal dystrophies encompass rod‐dominant diseases [such as retinitis pigmentosa (RP) or rod‐cone dystrophy (RCD), early onset retinal dystrophy (EORD), and Leber congenital amaurosis (LCA)] and cone‐dominant diseases (including cone/cone‐rod dystrophy, Stargardt disease, and macular dystrophies), with or without extraocular features. For those originating from the Arabian Gulf, approximately 5% of the population are affected with genetic disease involving the eye and adnexa (Tadmouri, Al‐Haj Ali, Nair, & Fareed, 2006), in comparison to just 0.0132% of children in the United Kingdom (Rahi & Cable, 2003). The most common mode of inheritance is autosomal recessive in 60%, autosomal dominant in 25%, X‐linked in 5%, and less than 1% are mitochondrial (Tadmouri et al., 2006). Endogamy and large families are a social preference in several Arab populations, and the consanguinity rate in the United Arab Emirates (UAE) is between 39 and 54.2% (Al‐Gazali & Hamamy, 2014). Together this contributes to the high number of cases with autosomal recessive diseases due to homozygous disease‐causing variants (Al‐Gazali & Hamamy, 2014; Tadmouri et al., 2006). Clinically accredited genetic testing permits the identification of disease‐causing gene variants and supports informed genetic counseling for family planning, potential therapies, and clinical trials (Prado, Acosta‐Acero, & Maldonado, 2020). In 2019 the UAE approved the use of Luxturna (or voretigene neparvovec), the first retinal gene therapy for patients with autosomal recessive *RPE65*‐retinopathy. We report herein the genetic outcomes for 74 unrelated families, with at least one member (proband) affected with nonsyndromic or syndromic inherited eye disease presenting consecutively to the ocular genetics service at Moorfields Eye Hospitals UAE over a 17‐month period from December 2017 to September 2019.

## 2 MATERIAL AND METHODS

A retrospective case note review of all consecutive patients presenting to the ocular genetics service at Moorfields Eye Hospitals UAE, Dubai and Abu Dhabi sites, from December 2017 to September 2019 was conducted. If the patient did not have a previously established genetic result, they were offered molecular testing using comparable targeted gene panel testing through the Rare & Inherited Disease Genomic Laboratory at Great Ormond Street Hospital (London, UK) or Blueprint Genetics (Helsinki, Finland). Coding exons and flanking intronic regions of genes associated with genetic eye diseases and selected deep intronic variants were screened and analyzed as previously reported (Patel et al., 2019). One proband (46‐1 from family 46) with aniridia initially had a microarray‐based comparative genomic hybridization for deletion screening of *WT1* and *PAX6* for Wilms tumor, aniridia, genitourinary anomalies, and mental retardation (WAGR) syndrome (OMIM #194072), this was negative, so then underwent *PAX6* (OMIM *607108) gene screening with Sanger sequencing through the Wessex Regional Genetics Laboratory (Salisbury, UK). Variant classification followed American College of Medical Genetics and Genomics (ACMG) guidelines (Richards et al., 2015). Pathogenic, likely pathogenic variants and variants with uncertain significance were confirmed by Sanger sequencing, if variants were consistent with the phenotype, the mode of inheritance, and familial history. The datasets (variants) generated from this study were submitted to ClinVar (https://www.ncbi.nlm.nih.gov/clinvar/) (SCV001335521–SCV001335530). All patients gave written informed consent for genetic testing. This study had local approval through Moorfields Eye Hospital and adhered to the tenets of the Declaration of Helsinki.

## 3 RESULTS

Ninety‐one patients from 74 unrelated families (with 74 probands), aged between 2 and 80 years old (mean ± *SD* was 27.4 ± 16.2 years), with 45% being female (41/91), presented to the ocular genetics service at Moorfields Eye Hospitals UAE. The ethnicity of families were Arab (74%, *n* = 55), Indian (8%, *n* = 6), Pakistani (7%, *n* = 5), Caucasian (5%, *n* = 4; Italian, British, South African, and Russian), Egyptian (3%; *n* = 2), Sudanese (1%, *n* = 1) and Japanese (1%, *n* = 1). Seventy‐eight percent of families (*n* = 58) reported consanguinity on direct questioning. Seven families presented with a predetermined genetic result and five families did not proceed with genetic testing due to the cost involved (Table S1); these 12 families were excluded from further analysis. Sixty‐two families proceeded with genetic testing to identify the gene variant(s) associated with their inherited eye disease using targeted gene panels, except one (family 46 with aniridia, as per the methods). All patient demographics including clinical and genetic details are listed in Table S1.

**FIGURE 1:**
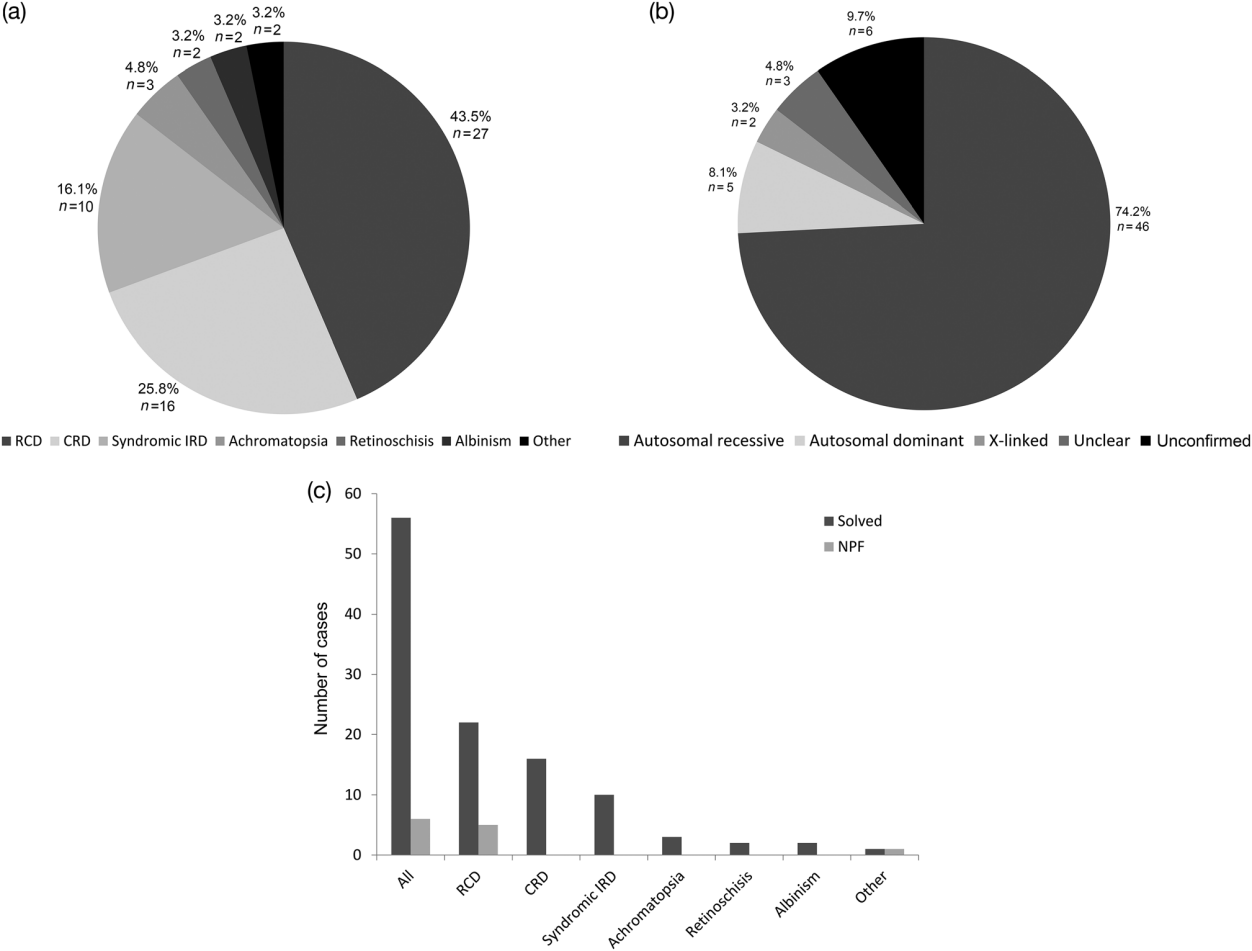
Disease subgroups and inheritance patterns of genetically solved and unsolved families. (a) Seventy‐four families from the UAE were grouped as follows: 27 rod‐cone dystrophies (RCD), 16 cone‐rod dystrophies (CRD), 10 syndromic retinal dystrophies (syndromic IRD), 3 achromatopsia, 2 retinoschisis, 2 albinism and 2 “others” (which include an aniridia and pathological myopia family). (b) Distribution of mode of inheritance amongst the 74 families. Three families remained “unconfirmed” with variants of unknown pathogenic significance or multiple mutations leading to uncertainty of the clinical diagnosis. NPF, no primary findings. (c) Number of families solved or with no primary finding (NPF) in each group

**FIGURE 2:**
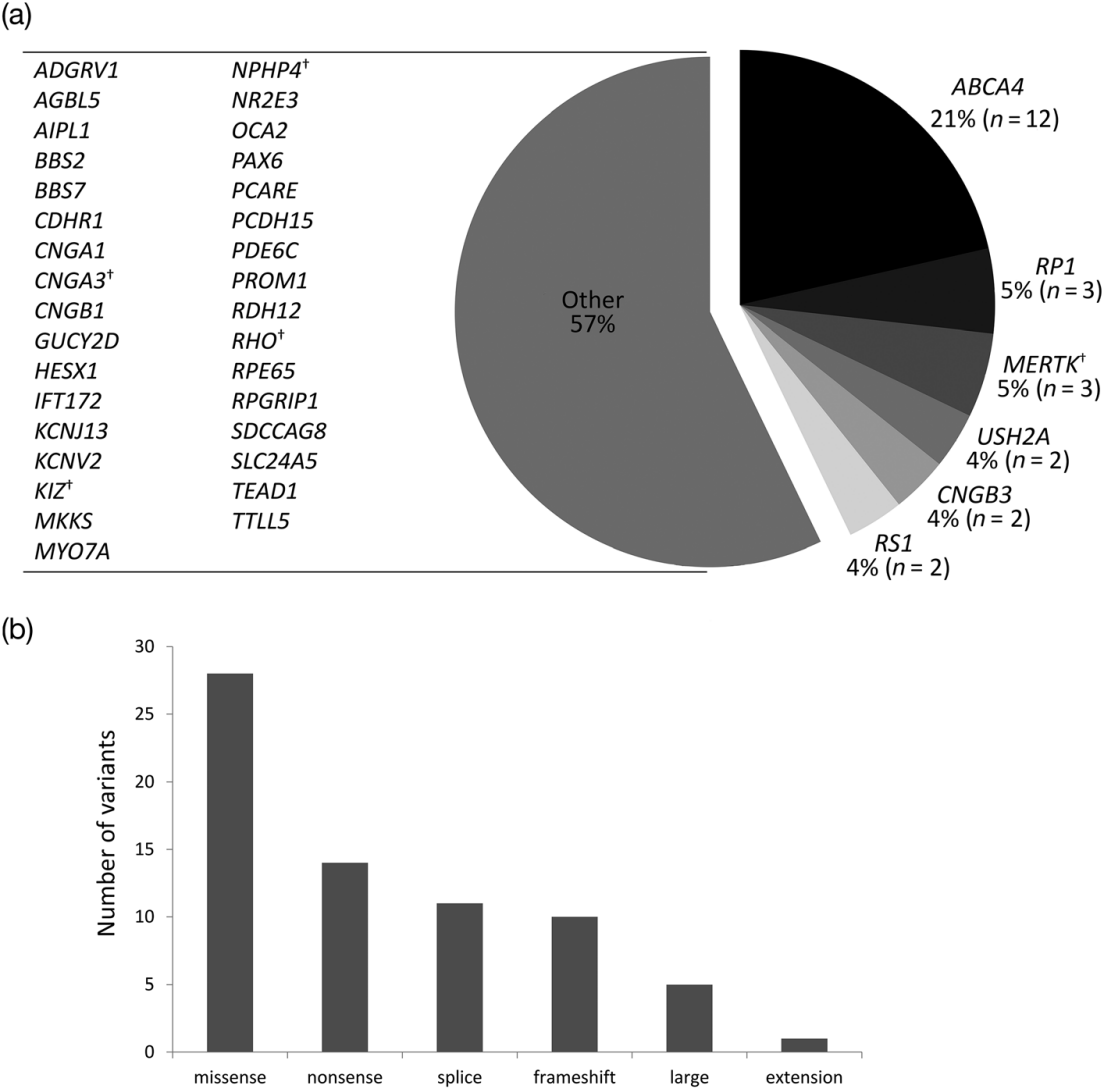
Representation of genes and variant subtypes associated with inherited eye disease in the molecularly confirmed 56 families. (a) Disease‐causative genes in this UAE cohort, the most common is *ABCA4*. Where a gene was identified in only one family from the entire cohort, it was placed in the “Other” group. ^†^Mutated variant(s) were found in this gene but the clinical diagnosis remains unconfirmed. (b) Types of variant identified within our cohort of families

Of the 62 families who opted for molecular testing, they were divided into 27 rod‐cone dystrophies (43.5%, including RP, EORD, LCA), 16 cone/cone‐rod dystrophies (25.8%, including Stargardt disease and macular dystrophies), 10 syndromic retinal dystrophies (16.1%), 3 achromatopsia (4.8%), 2 retinoschisis (3.2%), 2 albinism (3.2%) and 2 “others” (3.2%) including 1 aniridia and 1 pathological myopia (Figure 1a). The majority of conditions were inherited autosomal recessively with 46 affected families (74.2%), autosomal dominant in 5 families (8.1%), X‐linked recessive in 2 families (3.2%) and 3 with an unclear pattern of inheritance (4.8%) where the diagnosis remained unconfirmed (families 54, 55, and 56) (Figure 1b). In total, 56 families (90.3%) received a genetic diagnosis (Table 1; Figure 1c). Sixty‐nine variants were identified in 40 genes associated with inherited eye diseases (Table S2). The most prevalent gene was *ABCA4* found in 12 families, followed by *RP1* and *MERTK* in 3 families each, and then *USH2A*, *CNGB3* and *RS1* in 2 families each (Figure 2a). Among the 46 autosomal recessive families, 33 had homozygous variants (71.7%), the rest were compound heterozygous. A range of variants were identified with missense (*n* = 28), nonsense (*n* = 14), splice site (*n* = 11), frameshift (*n* = 10), large deletions or duplications (*n* = 5) and one novel extension [*ABCA4*: c.6820T>A p.(*2274Argext*34)] (Figure 2b). Several *ABCA4* variants were observed in multiple families: (a) Family 31 was homozygous for the complex allele c.5882G>A p.(Gly1961Glu)/c.2570T>C p.(Leu857Pro) considered to be a founder mutation; (b) Family 23 was compound heterozygous with this founder mutation; (c) Family 27 was heterozygous for the founder mutation and a further c.5882G>A p.(Gly1961Glu) variant; (d) Families 25 and 28 were compound heterozygous with c.5882G>A p.(Gly1961Glu) variant considered to be another founder mutation; and (5) the c.5714+5G>A variant was also observed in a heterozygous state in three families (families 22, 26, and 28). The *MERTK* deletion c.2214del p.(Cys738Trpfs*32) was reported in two families (families 11 and 12), one was in a homozygous state. The *USH2A* splice variant c.486‐1G>C was found to be homozygous in two families (40 and 41) (Table 1).

**TABLE 1:** Variant details and confirmed phenotype for the 56 solved families presenting to the ocular genetics service.

Family ID		Gene	Confirmed phenotype (OMIM#)	Zygosity	Variant	Variant type
1		*GUCY2D*	Leber congenital amaurosis 1 (204000)	Hom	c.3056A>C p.(His1019Pro)	Missense
2		*RPE65*	Leber congenital amaurosis 2 (204100)	Hom	c.1451‐2A>C	Splice site
3		*AIPL1*	Leber congenital amaurosis 4 (604393)	Het	c.404dup p.(Asp136Glyfs*22)	Frameshift
				Het	c.834G>A p.(Trp278*)	Nonsense
4		*RPGRIP1*	Leber congenital amaurosis 6 (613826)	Hom	c.1107del p.(Glu370Asnfs*5)	Frameshift
5		*RDH12*	Leber congenital amaurosis 13 (612712)	Hom	c.139G>A p.(Ala47Thr)	Missense
6		*KCNJ13*	Leber congenital amaurosis 16 (614186)	Hom	**c.431T>C p.(Leu144Pro)**	Missense
7		*RP1*	Retinitis pigmentosa 1 (180100)	Hom	c.1462del p.(Glu488Lysfs*44)	Frameshift
8		*RP1*	Retinitis pigmentosa 1 (180100)	Het	c.2219C>G p.(Ser740*)	Nonsense
9		*RP1*	Retinitis pigmentosa 1 (180100)	Het	c.310T>C p.(Tyr104His)	Missense
				Het	c.1047G>A p.(Trp349*)	Nonsense
10		*CRB1*	Retinitis pigmentosa 12 (600105)	Het	**c.2842+1delinsAA**	Splice site
				Het	c.2506C>A p.(Pro836Thr)	Missense
11		*MERTK*	Retinitis pigmentosa 38 (613862)	Hom	c.2214del p.(Cys738Trpfs*32)	Frameshift
12		*MERTK*	Retinitis pigmentosa 38 (613862)	Het	c.721C>T p.(Gln241*)	Nonsense
				Het	c.2214del p.(Cys738Trpfs*32)	Frameshift
13		*CNGB1*	Retinitis pigmentosa 45 (613767)	Het	c.973C>T p.(Gln325*)	Nonsense
				Het	**c.2977‐2del**	Splice site
14		*CNGA1*	Retinitis pigmentosa 49 (613756)	Hom	c.1035dup p.(Arg346Thrfs*7)	Frameshift
15		*PCARE*	Retinitis pigmentosa 54 (613428)	Hom	**c.3668+2T>C**	Splice site
16		*CDHR1*	Retinitis pigmentosa 65 (613660)	Hom	**Deletion of the first six coding exons**	Deletion
17		*IFT172*	Retinitis pigmentosa 71 (616394)	Hom	**c.1156C>T p.(Arg386Trp)**	Missense
18		*AGBL5*	Retinitis pigmentosa 75 (617023)	Hom	c.313_319del p.(Gly105Profs*24)	Frameshift
19		*TEAD1*	Sveinsson chorioretinal atrophy (108985)	Het	**Multi‐exon (1–8) duplication**	Duplication
20		*NR2E3*	Enhanced S‐cone syndrome (268100)	Hom	c.932G>A p.(Arg311Gln)	Missense
21		*ABCA4*	Stargardt disease 1 (248200)	Het	c.4793C>A p.(Ala1598Asp)	Missense
				Het	c.2382+4A>G	Splice site
22		*ABCA4*	Stargardt disease 1 (248200)	Het	c.5714+5G>A	Splice site
				Het	c.5461‐10T>C	Splice site
23		*ABCA4*	Stargardt disease 1 (248200)	Het	c.3898C>T p.(Arg1300*)	Nonsense
				Het	c.5882G>A p.(Gly1961Glu)	Missense
				Het	c.2570T>C p.(Leu857Pro)^a^	Missense
24		*ABCA4*	Stargardt disease 1 (248200)	Het	c.319C>T p.(Arg107*)	Nonsense
				Het	c.6380C>T p.(Ser2127Phe)	Missense
				Het	c.6148G>C p.(Val2050Leu)	Missense
25		*ABCA4*	Stargardt disease 1 (248200)	Het	c.1714C>T p.(Arg572*)	Nonsense
				Het	c.5882G>A p.(Gly1961Glu)	Missense
26		*ABCA4*	Stargardt disease 1 (248200)	Het	c.5714+5G>A	Splice site
				Het	c.1622T>C p.(Leu541Pro)	Missense
				Het	c.3113C>T p.(Ala1038Val)	Missense
27		*ABCA4*	Stargardt disease 1 (248200)	Hom	c.5882G>A p.(Gly1961Glu)	Missense
				Het	c.2570T>C p.(Leu857Pro)	Missense
28		*ABCA4*	Stargardt disease 1 (248200)	Het	c.5714+5G>A	Splice site
				Het	c.5882G>A p.(Gly1961Glu)	Missense
				Het	c.5512C>G p.(His1838Asp)	Missense
29		*ABCA4*	Stargardt disease 1 (248200)	Hom	c.6729+5_6729+19del p.(Phe2161Cysfs*3)	Frameshift
30		*ABCA4*	Stargardt disease 1 (248200)	Hom	c.1610G>A p.(Arg537His)	Missense
				Hom	c.6820T>A p.(*2274Argext*34)	Extension
31		*ABCA4*	Stargardt disease 1 (248200)	Hom	c.5882G>A p.(Gly1961Glu)	Missense
				Hom	c.2570T>C p.(Leu857Pro)	Missense
32		*ABCA4*	Stargardt disease 1 (248200)	Hom	**c.5137_5138delinsAG p.(Gln1713Arg)**	Missense
33		*PROM1*	Cone‐rod dystrophy 12 (612657)	Hom	c.1557C>G p.(Tyr519*)	Nonsense
34		*TTLL5*	Cone‐rod dystrophy 19 (615860)	Hom	**Multi‐exons (16–26) deletion**	Deletion
35		*KCNV2*	Retinal cone dystrophy 3B (610356)	Hom	c.427G>T p.(Glu143*)	Nonsense
36		*SDCCAG8*	Bardet–Biedl syndrome 16 (615993)	Hom	c.1444del p.(Thr482Leufs*12)	Frameshift
37		*HESX1*	Septooptic dysplasia (182230)	Het	**c.450C>G p.(Asp150Glu)**	Missense
38		*MYO7A*	Usher syndrome, type 1B (276900)	Hom	c.5392C>T p.(Gln1798*)	Nonsense
39		*PCDH15*	Usher syndrome, type 1F (602083)	Hom	Deletion of the first three coding exons	Deletion
40		*USH2A*	Usher syndrome, type 2A (276901)	Hom	c.486‐1G>C	Splice site
41		*USH2A*	Usher syndrome, type 2A (276901)	Hom	c.486‐1G>C	Splice site
42		*ADGRV1*	Usher syndrome, type 2C (605472)	Hom	c.12798T>A p.(Tyr4266*)	Nonsense
43		*BBS2*	Bardet–Biedl syndrome 2 (615981)	Hom	c.117G>A p.(Lys39=)	Synonymous, splice site
44		*MKKS*	Bardet–Biedl syndrome 6 (605231)	Hom	c.295T>C p.(Cys99Arg)	Missense
45		*BBS7*	Bardet–Biedl syndrome 7 (615984)	Hom	c.968A>G p.(His323Arg)	Missense
46		*PAX6*	Aniridia (106210)	Het	c.107_114dup p.(Pro39Glyfs*18)	Frameshift
47		*CNGB3*	Achromatopsia 3 (262300)	Hom	c.1148del p.(Thr383Ilefs*13)	Frameshift
48		*CNGB3*	Achromatopsia 3 (262300)	Hom	c.1063C>T p.(Arg355*)	Nonsense
49		*PDE6C*	Cone dystrophy 4 (613093)	Hom	c.490T>C p.(Phe164Leu)	Missense
50		*OCA2*	Albinism, oculocutaneous, type II (203200)	Het	**c.890+1G>A**	Splice site
51		*SLC24A5*	Albinism, oculocutaneous, type VI (113750)	Hom	c.328G>C p.(Gly110Arg)	Missense
52		*RS1*	Retinoschisis (312700)	Hemi	c.305G>A p.(Arg102Gln)	Missense
53		*RS1*	Retinoschisis (312700)	Hemi	c.304C>T p.(Arg102Trp)	Missense
54		*NPHP4* ^b^	Senior‐Løken 4 (606996)	Hom	c.955A>G p.(Ser319Gly)	Missense
55		*MERTK* ^b^	Retinitis pigmentosa 38 (613862)	Het	Multi‐exon (3–19) deletion	Deletion
				Het	c.845‐18G>A	Splice site
56		*KIZ* ^b^	Retinitis pigmentosa 69 (615780)	Het	c.583C>T p.(Arg195*)	Nonsense
		*CNGA3* ^b^	Achromatopsia 2 (216900)	Het	c.967G>C p.(Ala323Pro)	Missense
				Het	c.1705C>T p.(Arg569Cys)	Missense
		*RHO* ^b^	Night blindness, congenital stationary, autosomal dominant (610445) Retinitis pigmentosa 4, autosomal dominant or recessive (613731)	Het	c.70T>C p.(Phe24Leu)	Missense

Each variant was confirmed by Sanger sequencing. Endpoints of large deletion or duplications could not be defined with the targeted gene panel approach. Fourteen novel variants in twelve genes, with three large duplications or deletions in three genes, are depicted in bold.

Abbreviations: Hemi, hemizygous; Het, heterozygous; Hom, homozygous.

^a^ Not confirmed by Sanger.

^b^ Variant(s) found but the diagnosis is unconfirmed.

**FIGURE 3:**
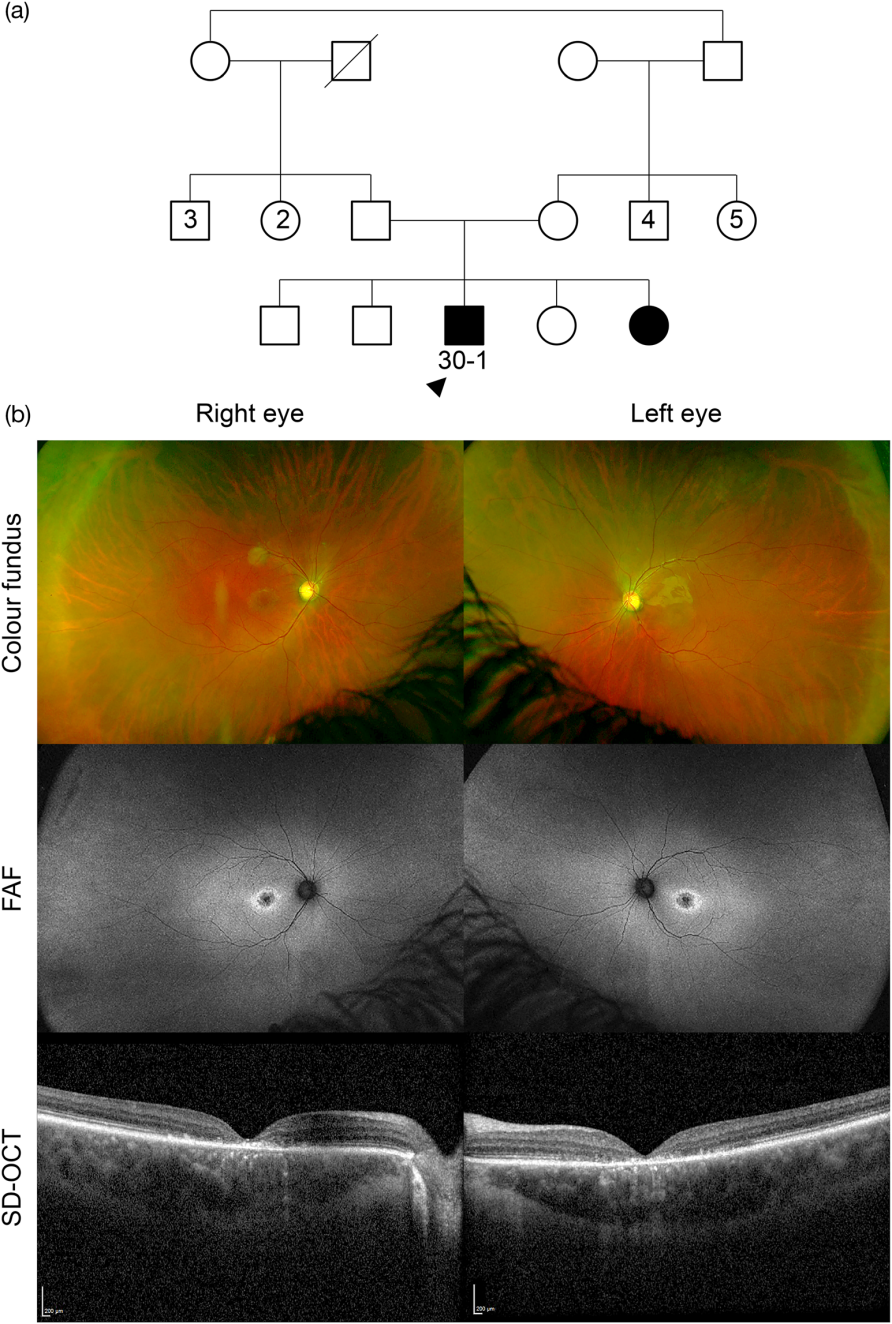
Family 30 has an *ABCA4*‐associated retinopathy with homozygous complex variants c.1610G>A p.(Arg537His)/c.6820T>A p.(*2274Argext*34). (a) Family tree highlighting the proband (30‐1, arrowhead) and his affected sister with parental consanguinity. (b) The clinical phenotype in proband 30‐1 shows a bilateral area of central macular atrophy on the ultra‐widefield (UWF) color fundus images with associated yellow macular flecks. Fundus autofluorescence (FAF) revealed increased macular autofluorescence with a central hypoautofluorescence in the area of macular atrophy, surrounded with a ring of hyperautofluorescence corresponding with the yellow flecks. Spectral‐domain optical coherence tomography (SD‐OCT) with a horizontal line scan through the foveola shows loss of outer retinal structures and ellipsoid zone in both eyes

Fourteen novel variants were detected with 2 large deletions in *CDHR1* (family 16) and *TTLL5* (family 34), a multi‐exon (exons 1–8) duplication in *TEAD1* (family 19) and 11 single nucleotide and small insertion/deletion variants in nine further genes were found in this study; *ABCA4*, *CNGB1*, *KCNJ13*, *CRB1*, *IFT172*, *AGBL5*, *PCARE*, *HESX1* and *OCA2* (Table 1). Family 30 was homozygous for variants c.1610G>A p.(Arg537His) and c.6820T>A p.(*2274Argext*34) in *ABCA4*. The proband (30‐1) was a 22‐year‐old male who reported difficulty reading the classroom board at school from the age of 6 years, his central vision deteriorated slowly over time with his contrast and color vision, and he complained of photophobia. No other systemic features or past medical history. His family is consanguineous, and he has a younger affected sister age 14. His best corrected visual acuity (BCVA) using LogMAR was 0.82 in the right eye and 0.90 in the left eye, normal intraocular pressure, fundus examination revealed bilateral central macular atrophy associated yellow macular flecks. Fundus autofluorescence (FAF) revealed increased macular autofluorescence with a central hypoautofluorescence in the area of macular atrophy, surrounded with a ring of hyperautofluorescence corresponding with the yellow flecks. Spectral‐domain optical coherence tomography (SD‐OCT) with a horizontal line scan through the foveola shows loss of outer retinal structures and ellipsoid zone in both eyes (Figure 3).

**FIGURE 4:**
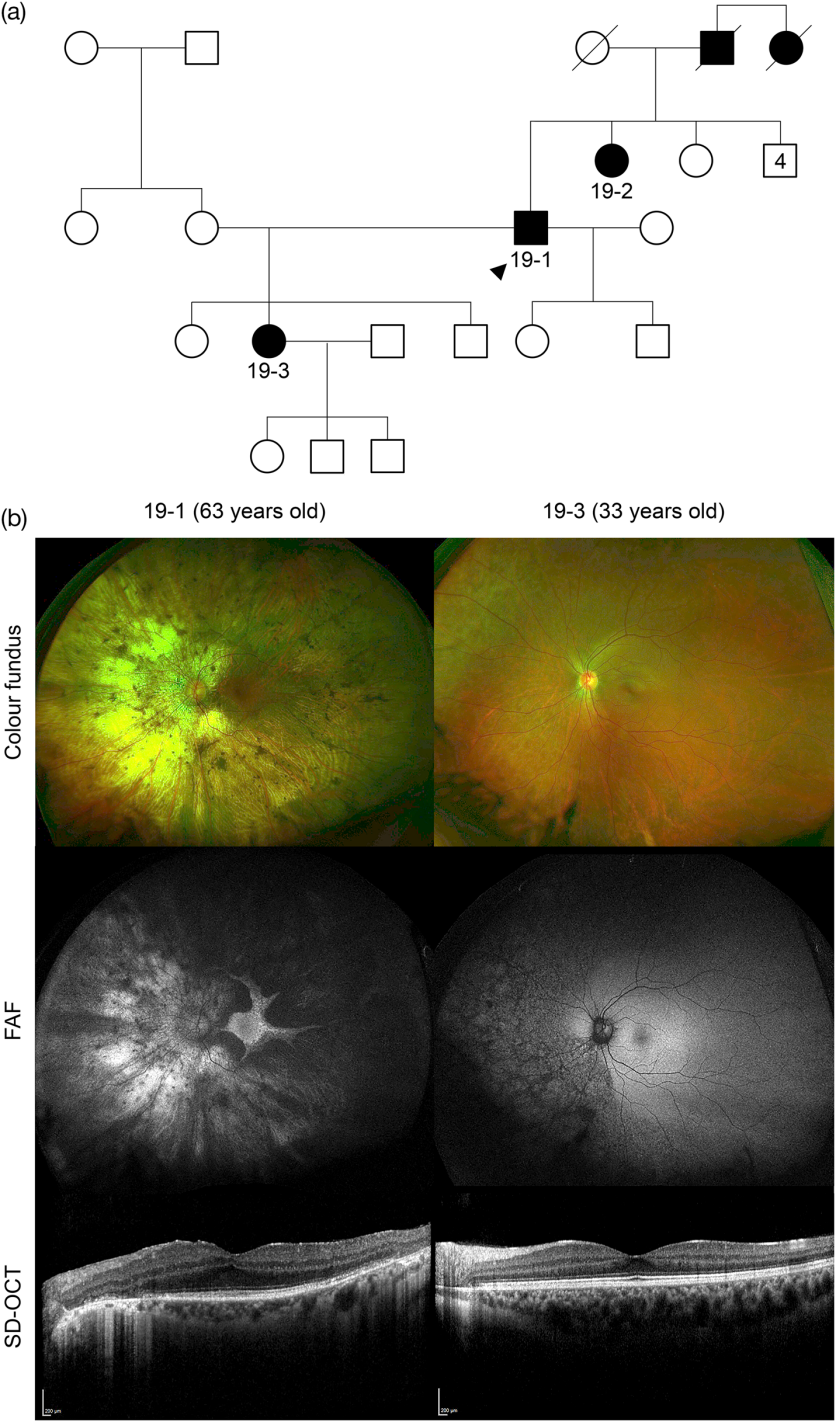
Family 19 has a novel heterozygous multi‐exon (1–8) duplication of *TEAD1*, associated with Sveinsson chorioretinal atrophy. (a) Family tree highlighting the autosomal dominant inheritance, no consanguinity, with proband (19‐1, arrowhead), his affected sister (19‐2), daughter (19‐3) and deceased father and paternal aunt. (b) The clinical phenotype of the left eye is shown (right eye had symmetrical findings) of the proband 19‐1 aged 63 years and his affected daughter (19‐3) aged 33 years. In proband 19‐1, there is an extensive widespread chorioretinal atrophy, more marked on the nasal side and peripapillary region, with a preserved central macular retinal island on the UWF color fundus imaging. FAF imaging shows a well delineated hyperautofluorescent retinal island with scalloped edges, the superotemporal retina has hypoautofluorescence. SD‐OCT with a horizontal line scan through the fovea shows relatively well‐preserved ellipsoid zone with clear edges of outer retinal layer disruption and loss. In patient 19‐3, there are RPE changes extending from inferonasal peripapillary margin to the far nasal retina, corresponding to changes in the FAF, which shows scalloped hypoautofluorescence throughout this area. SD‐OCT shows a healthy macula with intact ellipsoid zone

Family 19 were found to have a novel heterozygous multi‐exon (exons 1–8) duplication in *TEAD1* displaying a clinical phenotype consistent with Sveinsson chorioretinal atrophy. The proband (19‐1), now 63 years old, noticed difficulties with his night vision and peripheral visual field when he was age 30 years, he was diagnosed with RP and primary acute angle closure for which he had a left yag laser peripheral iridectomy (PI). He had a right phaco and IOL for cataract extraction age 54 and has a left cataract *in situ*. His color vision is normal but over the past 5 years the nyctalopia has worsened. No systemic features, a past medical history of prostate cancer, but nil else of note. The family is non‐consanguineous, but shows a dominant inheritance with an affected daughter (19‐3) who is 33 years old, an affected older sister (192) who is 67 years old, his late father and paternal aunt were also affected (see Figure 4a). On examination, BCVA of 0.82 in the right eye and 0.90 in the left eye, intraocular pressure was normal. Anterior segment showed right pseudophakia, left superior PI, and mixed cortical and nuclear cataract. Fundus examination revealed bilateral peripapillary chorioretinal atrophy, with the nasal retina significantly affected extending to the periphery, and a preserved central macular island consistent with an appearance of advanced helicoidal peripapillary chorioretinal degeneration (Figure 4b). FAF imaging showed a well‐delineated hyperautofluorescent retinal island with scalloped edges, and the superotemporal retina has hypoautofluorescence. SD‐OCT with a horizontal line scan through the fovea shows relatively well‐preserved ellipsoid zone with clear edges of outer retinal layer disruption and loss. His daughter (19‐3), had a BCVA of LogMAR 0.00 in both eyes, but showed early signs of the disease with RPE changes in the nasal retina extending from the inferonasal peripapillary region (Figure 4b). Her central macula was unaffected by the disease at this stage as seen with the FAF and OCT imaging.

## 4 DISCUSSION

Herein, we report the genetic outcomes of 74 consecutive families affected with inherited eye diseases based in the UAE presenting over 17 months. The applied targeted gene panel approach provided 90.3% of families (56/62 tested) with a molecular diagnosis. The cone‐rod dystrophy subgroup were predominantly associated with *ABCA4* variants, unlike the rod‐cone dystrophy group that had a more heterogeneous representation of 16 different genes. All syndromic retinal dystrophy families were solved, but in some, the key syndromic features were not reported and further investigations were required. For example, in family 36, a homozygous deletion c.1444del p.(Thr482Leufs*12) in *SDCCAG8* was identified in proband 36‐1 age 10, this gene is known to cause Senior‐Løken syndrome 7 (OMIM #613615) and Bardet–Biedl syndrome 16 (OMIM #615993), two multisystem ciliopathies with RP. This patient initially presented with RP and an intermittent alternating exotropia, hearing impairment, and recurrent bronchitis, but no other past medical history (including no polydactyly, obesity, learning difficulties, or renal impairment). They were referred to pediatrics for renal assessment of nephronophthisis and to investigate for any other syndromic features. A previous report of an Indian patient, no details of age or gender were provided, harboring the c.1444del p.(Thr482Leufs*12) variant with a c.1627_1630del p.(Asp543fs*566) was diagnosed with Bardet–Biedl syndrome 16, and displayed RP, obesity, nephronophthisis, end stage kidney failure, and mild intellectual disability, but absence of polydactyly (Otto et al., 2010). One other family within this series had four Gypsy siblings but reported a relatively late onset of renal and retinal disease in their twenties with mild intellectual disability and obesity. They were homozygous for the c.704+365C>T variant, which leads to frameshift mutation. The small amount of protein may explain the reported clinical variability. Two Indian sisters, aged 16 and 13 years, were also reported harboring the same genotype [c.1444del p.(Thr482Leufs*12); c.1627_1630del p.(Asp543fs*566)], associated with Bardet–Biedl syndrome 16 but had end stage renal disease at 11 and 9 years, respectively, without polydactyly (Billingsley, Vincent, Deveault, & Héon, 2012). Genetic modifiers may explain the variability of onset and severity in these patients (Meyer & Anderson, 2017).

**FIGURE 5:**
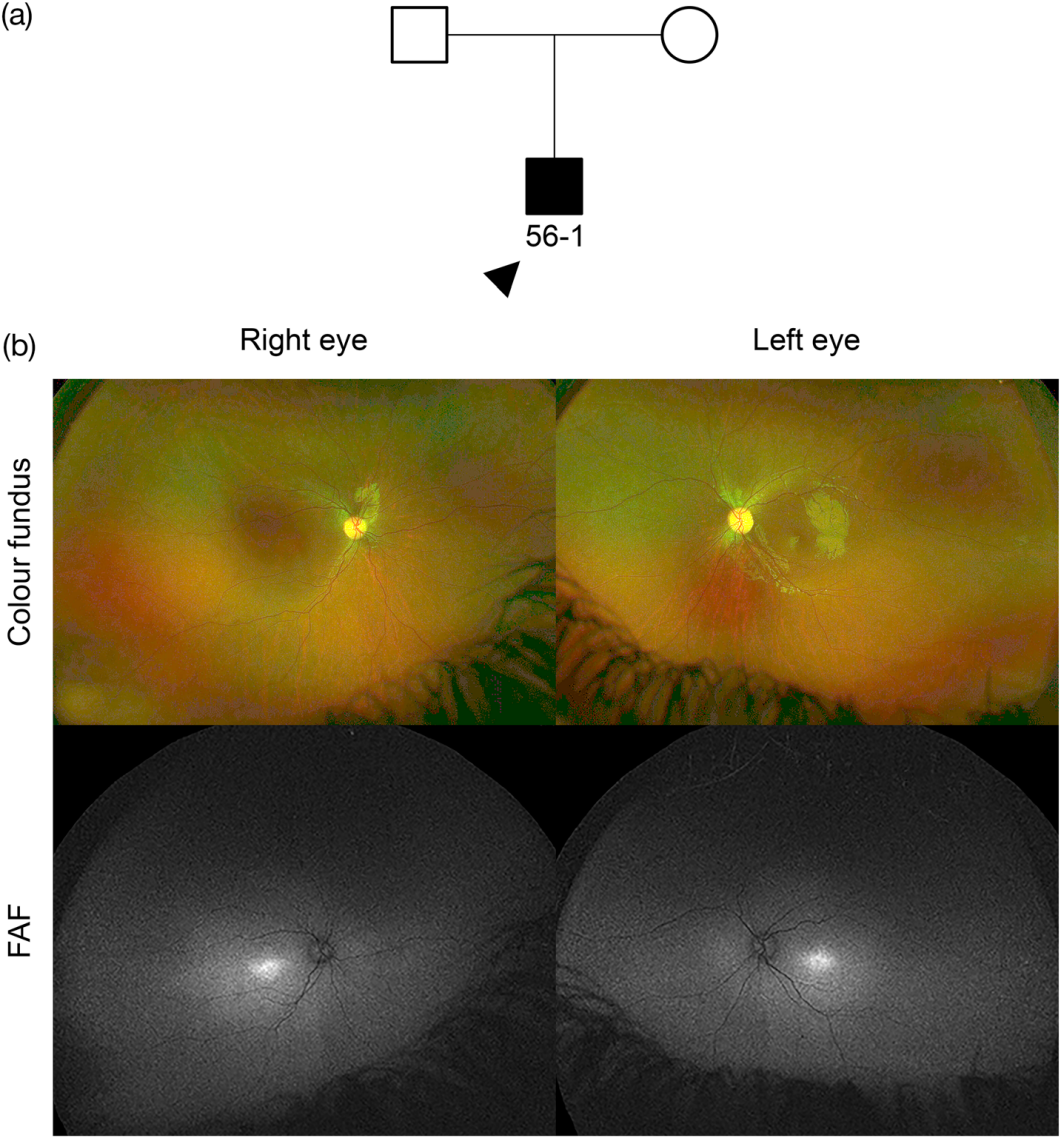
Family 56 has multiple variants in *CNGA3*, *KIZ*, and *RHO* (see Table 1 for details). (a) Family tree highlighting the proband (56‐1, arrowhead), no reported consanguinity. (b) The clinical phenotype in proband 56‐1 aged 9 years shows a normal fundus appearance on the UWF color fundus images. UWF FAF revealed foveal hyperautofluorescence. SD‐OCT was not available for this patient due to their nystagmus. The clinical features appear consistent with *CNGA3*‐related achromatopsia

Despite the high rates of molecular diagnosis, 9.7% of families remained without any primary findings; the largest group were the rod‐cone dystrophies with five unsolved families. In the “other” group, one family 69 with pathological myopia, remained unsolved. Three families (54, 55, and 56) had putative disease‐causing variants of unknown pathogenic significance. In family 54, the proband 54‐1 was a 34‐year‐old male who reported reduced distance vision from the age of 27 and mild nyctalopia, no other systemic features or past medical history. He has a consanguineous family with an affected older brother, age 37 years, who developed visual symptoms from age 14, affected mother, maternal uncle and maternal grandfather. BCVA with LogMAR was 0.00 in the right eye and −0.08 in the left eye, normal intraocular pressure and anterior segment examination revealed mild posterior subcapsular cataracts in both eyes. Fundus examination revealed healthy optic discs, mild retinal vessel attenuation, bone spicules in the mid‐periphery with RPE changes, and fine white flecks at the macula. He was found to have a novel homozygous missense variant c.955A>G p.(Ser319Gly) in *NPHP4*, which causes Senior‐Løken syndrome 4 (OMIM #606996). There have been no reports of non‐syndromic retinal dystrophy, and missense mutations have been found to cause syndromic disease, hence this patient has also been referred for further renal investigation. Renal abnormalities can have a variable range of onset in Senior‐Løken syndrome, as reported for patients with *IQCB1*‐related retinal dystrophy where nephronophthisis and end‐stage renal disease commences between age 3 and 50 years (Estrada‐Cuzcano et al., 2011). In family 55, a compound heterozygous multi‐exon (3–19) deletion and a splice site variant c.845‐18G>A (within this deleted area) was found in *MERTK*. The splice variant frequency was 0.068% in the worldwide population (gnomAD), more frequent in the African population (0.7%), and the impact on splicing remains unconfirmed. Further analysis is needed to study the splice effect and to delineate the multi‐exon deletion breakpoints. A deep intronic *MERTK* variant or mutation in a regulatory sequence not covered by the panel could not be excluded. Whole genome sequencing (WGS) can cover deep intronic, 3′‐ and 5′‐untranslated regions and noncoding regulatory elements, whilst also covering novel genes, and is beneficial for unsolved cases. In family 56, multiple disease‐causing variants were identified leaving the molecular diagnosis inconclusive until further clinical investigations such as electroretinography (ERG) or WGS is undertaken. Proband 56‐1 was a 9‐year‐old male presenting with nystagmus from birth and reduced vision from age 1 with photophobia and a hypermetropic astigmatism, no other systemic features or past medical history, and the family are non‐consanguineous. BCVA with LogMAR was 1.10 in the right eye and 1.00 in the left eye, anterior segment, and fundus examination was normal, without any abnormalities such as pigmentary or atrophic changes, and FAF showed bilateral foveal hyperautofluorescence (Figure 5). The following variants were identified and confirmed by Sanger sequencing: (a) heterozygous *CNGA3* variants c.967G>C p.(Ala323Pro) and c.1705C>T p.(Arg569Cys), which can cause achromatopsia 2 (OMIM #216900) (Nishiguchi, Sandberg, Gorji, Berson, & Dryja, 2005); (b) a heterozygous *RHO* variant c.70T>C p.(Phe24Leu), very close to the most frequent autosomal dominant variant p.Pro23His (Dryja et al., 1990) associated with retinitis pigmentosa 4 (OMIM #613731); and (c) a heterozygous nonsense variant c.583C>T p.(Arg195*) in *KIZ*, which causes autosomal recessive retinitis pigmentosa 69 (OMIM #615780) (El Shamieh et al., 2014). In this case, the retinal appearance was in keeping with *CNGA3*‐related achromatopsia, however, an ERG is required to determine whether there are diminished or absent photopic responses, with normal scotopic responses, compared with those seen for RP. Familial segregation and possible WGS to ascertain a second possible *KIZ* variant should be considered. This is a complex case of possible dual retinal pathology, as observed in a Senegalese man affected with *CC2D2A*‐related rod‐cone dystrophy and *CNGA3*‐related achromatopsia (Méjécase et al., 2019). The case of proband 56‐1 highlights a dilemma for future therapeutic intervention especially in view of the current achromatopsia *CNGA3* gene therapy trials [NCT 02610582 (Reichel et al., 2017), 03278873, 03758404, and 02935517].

Novel variants often require further evidence to support their pathogenicity but careful clinical phenotyping with the use of Human Phenotype Ontology (HPO) terms can support a molecular diagnosis. For example in family 19, a novel multiexon duplication in *TEAD1* was detected with an associated clinical phenotype consistent with Sveinsson chorioretinal atrophy (OMIM #108985, Figure 4). Only a missense variant c.1261T>C p.(Tyr421His) has been associated with this condition previously (Fossdal et al., 2004). In family 6, two affected patients 6‐1 (8 years old) and 6‐2 (4 years old) with a severe EORD were homozygous for a novel missense variant c.431T>C p.(Leu144Pro) in *KCNJ13*, predicted to be disease‐causing and found only twice in a heterozygous state in 125,455 people (allele frequency = 0.0007971%) from multiple origins (gnomAD). This variant is localized to the conserved transmembrane M2 protein domain, close to the missense variant c.458C>T p.(Thr153Ile) previously reported to cause Leber congenital amaurosis 16 (OMIM #614186) with retinovascular changes in late adolescence. The fundal appearance was consistent with *KCNJ13*‐retinopathy and regular fundus examinations are required to mitigate any signs of neovascularization with age (Toms et al., 2019).

*ABCA4* variants are amongst the most common in the UAE cohort (Burke et al., 2012; Guymer et al., 2001; Khan, 2019a, 2019b). Family 30 had a 22‐year‐old male proband (30‐1) with a clinical diagnosis of Stargardt disease. He was found to have a homozygous complex variant c.1610G>A p.(Arg537His)/c.6820T>A p.(*2274Argext*34). The c.1610G>A p.(Arg537His) variant has been previously reported to be associated with a complex allele [c.1622T>C p.(Leu541Pro) and c.3113C>T p.(Ala1038Val)] in a patient with typical Stargardt disease (Avela et al., 2018). The variant c.6820T>A p.(*2274Argext*34) is novel, absent from gnomAD and affects the last codon leading to an extension of the ABCA4 protein. The two homozygous mutations (missense and extension) together did not result in a severe phenotype as expected (Figure 3).

Variants in *ABCA4*, *MERTK* and *KCNV2* have previously been suggested as founder mutations in the UAE population (Khan, 2019b). In our cohort, several of these variants were identified providing further evidence for this: (a) *MERTK* deletion c.2214del p.(Cys738Trpfs*32) was reported in two unrelated families (family 12 and homozygous in family 11); (b) *KCNV2* nonsense variant c.427G>T p.(Glu143*) was reported in one family (family 34) in the homozygous state. For *ABCA4*, 5 families (families 23, 25, 27, 28, 31; in a homozygous state in families 27 and 31), all of Arab Emirati descent shared the variant c.5882G>A p.(Gly1961Glu), which has been reported as founder mutation in those of Somalian origin (Burke et al., 2012; Guymer et al., 2001). The c.5882G>A p.(Gly1961Glu) variant has been associated in *cis* with c.2570T>C p.(Leu857Pro) and suggested to be the UAE founder mutation (Khan, 2019b). This was seen in three families (23, 27, and 31) in association with a further heterozygous change, but in family 31 both variants were found in a homozygous arrangement (Table 1). The variant frequency of c.5882G>A p.(Gly1961Glu) was 5.9% (5/85 individuals) in this cohort compared with 0.46% in the worldwide population (gnomAD). A further *ABCA4* splice variant c.5714+5G>A was observed in a compound heterozygous state in three unrelated families (22, 26, and 28), this is considered a “mild” variant also found in those of European descent and whose frequency has increased in Newfoundland, Canada due to a founder effect (Green et al., 2020). A larger UAE molecularly confirmed cohort will further support the evidence for founder mutations, which may be abundant in this population due to the high consanguinity rate.

Amongst the 46 families with autosomal recessive inherited eye disease, homozygous variants were identified in 71.7% of cases. In the 42 reported consanguineous families, 32 had homozygous variants (74%). The consanguinity rate in this cohort was 78%, whereas in other studies, it ranged from 39 to 54.2% in the UAE (Al‐Gazali & Hamamy, 2014). It is important to provide sensitive genetic counseling to these families, explaining the inheritance patterns and the result of consanguinity, with the risk of further affected children and significance for future generations.

In this study we report the molecular diagnosis of 56 unrelated families originating or residing in the UAE. The majority of patients presenting to the clinic were affected with inherited retinal disease, highlighting a dearth of other nonretinal genetic eye conditions such as primary congenital glaucoma, congenital cataract, and ocular malformations. This may reflect referring practice and a lack of awareness relating to genetic testing for these conditions as extensive nonretinal targeted gene panels covering these conditions exists that can be offered to families. Patients who presented for genetic testing had a high diagnostic yield, but in some cases the number of pathogenic variants due to consanguinity confound the overall retinal pathology and make therapeutic choices difficult. The visual prognosis in these patients is variable. Nonetheless, for the majority it will guide eligibility into clinical trials and future approved therapies such as voretigene neparvovec, which is now available in the region. Further large‐scale studies in the UAE population will reveal founder mutations associated with inherited eye diseases due to the engrained endogamy and consanguinity. Those who remain with no primary findings or inconclusive results will benefit from whole genome sequencing, which will become the gold‐standard genetic test for all patients in the future.

## Supporting information

**TABLE S1** All demographic, clinical and genetic details from 74 consecutive families with inherited eye disease presenting to the ocular genetics service at Moorfields Eye Hospitals UAE between December 2017 and September 2019. Abbreviations: AD, autosomal dominant; AR, autosomal recessive; CRD, cone‐rod dystrophy; EORD, early onset retinal dystrophy; F, female; LCA, Leber congenital amaurosis; M, male; NPF, no primary finding; RP, retinitis pigmentosa; XL, X‐linked. ^†^Variant(s) found but the diagnosis is unconfirmed. ^‡^Patient attended clinic with already established molecular diagnosis (genetic testing performed elsewhere).

**TABLE S2** Each variant identified in this cohort is listed below according to the American College of Medical Genetics and Genomics (ACMG) 2015 criteria (Richards et al., 2015). Fourteen novel variants in twelve genes, with three large duplications or deletions in three genes, are depicted in bold. ^†^Variant(s) found but the diagnosis remains unconfirmed.
