# Supplementary material for: The genetic landscape of inherited eye disorders in 74 consecutive families from the United Arab Emirates
Source: Am J Med Genet C Semin Med Genet. 2020 Aug 11;184(3):762–72. doi: 10.1002/ajmg.c.31824 (PMC8432150; doi:10.1002/ajmg.c.31824)
Supplement: Supplementary file 1 — TABLE S1 All demographic, clinical and genetic details from 74 consecutive families with inherited eye disease presenting to the ocular genetics service at Moorfields Eye Hospitals UAE between December 2017 and September 2019. Abbreviations: AD, autosomal dominant; AR, autosomal recessive; CRD, cone‐rod dystrophy; EORD, early onset retinal dystrophy; F, female; LCA, Leber congenital amaurosis; M, male; NPF, no primary finding; RP, retinitis pigmentosa; XL, X‐linked. †Variant(s) found but the diagnosis is unconfirmed. ‡Patient attended clinic with already established molecular diagnosis (genetic testing performed elsewhere). [file AJMG-184-762-s002.docx]

**Supplementary table 1:** All demographic, clinical and genetic details from 74 consecutive families with inherited eye disease presenting to the ocular genetics service at Moorfields Eye Hospitals UAE between December 2017 to September 2019. Abbreviations: M: male; F: female. EORD: early onset retinal dystrophy; RP: retinitis pigmentosa; LCA: Leber congenital amaurosis; CRD: cone-rod dystrophy. AD: autosomal dominant. AR: autosomal recessive. XL: X-linked. NPF: no primary finding. ^†^Variant(s) found but the diagnosis is unconfirmed. ^‡^Patient attended clinic with already established molecular diagnosis (genetic testing performed elsewhere).

| **Family ID** | **Patient ID** | **Age (years)** | **Gender** | **Ethnicity** | **Consanguinity** | **Clinical phenotype** | **Inheritance** | **Gene** |
| --- | --- | --- | --- | --- | --- | --- | --- | --- |
| 1 | 1-1 | 11 | F | Arab | Yes | LCA, cataracts (from eye-poking) | AR | *GUCY2D* |
| 2 | 2-1 | 22 | M | Arab | Yes | LCA | AR | *RPE65* |
| 3 | 3-1 | 17 | M | Arab | Yes | LCA, nystagmus | AR | *AIPL1* |
| 3 | 3-2 | 10 | F | Arab | Yes | LCA, nystagmus, hypermetropia | AR | *AIPL1* |
| 4 | 4-1 | 17 | M | Arab | Yes | LCA, nystagmus, bilateral keratoconus, white cataract | AR | *RPGRIP1* |
| 5 | 5-1 | 14 | F | Arab | Yes | EORD, nystagmus, bilateral hypermetropia | AR | *RDH12* |
| 5 | 5-2 | 12 | M | Arab | Yes | EORD, nystagmus, bilateral hypermetropia | AR | *RDH12* |
| 6 | 6-1 | 8 | F | Arab | Yes | EORD, nystagmus | AR | *KCNJ13* |
| 6 | 6-2 | 4 | F | Arab | Yes | EORD, nystagmus, exotropia, myopia | AR | *KCNJ13* |
| 7 | 7-1 | 22 | F | Arab | Yes | EORD, high myopia | AR | *RP1* |
| 8 | 8-1 | 45 | F | Caucasian Italian | No | RP, bilateral irregular astigmatism | AD | *RP1* |
| 9 | 9-1 | 29 | M | Arab | Yes | RP | AR | *RP1* |
| 9 | 9-2 | 25 | M | Arab | Yes | RP | AR | *RP1* |
| 10 | 10-1 | 28 | M | Arab | Yes | RP | AR | *CRB1* |
| 11 | 11-1 | 47 | F | Arab | Yes | RP | AR | *MERTK* |
| 11 | 11-2 | 40 | M | Arab | Yes | RP | AR | *MERTK* |
| 12 | 12-1 | 17 | F | Arab | Yes | RP | AR | *MERTK* |
| 12 | 12-2 | 20 | M | Arab | Yes | RP | AR | *MERTK* |
| 13 | 13-1 | 53 | F | Caucasian; South African | No | RP | AR | *CNGB1* |
|  |  |  |  |  |  |  |  |  |
| 14 | 14-1 | 28 | F | Indian | Yes | EORD | AR | *CNGA1* |
| 15 | 15-1 | 28 | F | Arab | Yes | RP | AR | *PCARE* |
| 16 | 16-1 | 34 | M | Arab | Yes | RP | AR | *CDHR1* |
| 17 | 17-1 | 51 | F | Arab | Yes | RP | AR | *IFT172* |
| 17 | 17-2 | 54 | M | Arab | Yes | RP, bilateral posterior subcapsular cataract | AR | *IFT172* |
| 18 | 18-1 | 28 | M | Arab | Yes | RP, myopia | AR | *AGBL5* |
| 19 | 19-1 | 63 | M | Arab | No | RP, nuclear cataract, primary angle-closure glaucoma | AD | *TEAD1* |
| 19 | 19-2 | 67 | F | Arab | No | RP | AD | *TEAD1* |
| 19 | 19-3 | 33 | F | Arab | No | RP, Left corneal dermoid | AD | *TEAD1* |
| 20 | 20-1 | 17 | F | Arab | Yes | Enhanced S-cone syndrome | AR | *NR2E3* |
| 21 | 21-1 | 18 | M | Arab | Yes | Stargardt disease | AR | *ABCA4* |
| 21 | 21-2 | 14 | F | Arab | Yes | Stargardt disease | AR | *ABCA4* |
| 22 | 22-1 | 45 | F | Arab | No | Stargardt disease | AR | *ABCA4* |
| 23 | 23-1 | 35 | F | Arab | Yes | Stargardt disease | AR | *ABCA4* |
| 24 | 24-1 | 41 | F | Caucasian British | No | Stargardt disease | AR | *ABCA4* |
| 25 | 25-1 | 25 | M | Arab | Yes | Bull’s eye maculopathy | AR | *ABCA4* |
| 26 | 26-1 | 37 | F | Caucasian; Russian | No | Stargardt disease | AR | *ABCA4* |
| 27 | 27-1 | 31 | M | Arab | Yes | Stargardt disease | AR | *ABCA4* |
| 28 | 28-1 | 35 | F | Arab | Yes | Stargardt disease | AR | *ABCA4* |
| 29 | 29-1 | 28 | M | Indian | No | CRD | AR | *ABCA4* |
| 30 | 30-1 | 22 | M | Arab | Yes | Stargardt disease | AR | *ABCA4* |
| 31 | 31-1 | 14 | M | Arab | Yes | Stargardt disease | AR | *ABCA4* |
| 32 | 32-1 | 32 | M | Arab | Yes | Stargardt disease | AR | *ABCA4* |
| 33 | 33-1 | 8 | M | Arab | Yes | EORD, nystagmus | AR | *PROM1* |
| 34 | 34-1 | 16 | F | Arab | Yes | EORD, tilted discs | AR | *TTLL5* |
| 35 | 35-1 | 19 | F | Arab | Yes | Bull’s eye maculopathy | AR | *KCNV2* |
| 35 | 35-2 | 20 | F | Arab | Yes | Bull’s eye maculopathy, astigmatism, myopia | AR | *KCNV2* |
| 36 | 36-1 | 10 | M | Indian | No | RP, intermittent alternating exotropia, hearing impairment, recurrent bronchitis | AR | *SDCCAG8* |
| 37 | 37-1 | 22 | F | Arab | Yes | Optic nerve hypoplasia, cataracts, with obesity, syndactyly, dental anomalies, thyroid dysfunction, learning difficulties | AD | *HESX1* |
| 38 | 38-1 | 14 | F | Arab | Yes | Usher syndrome, sensorineural congenital hearing loss and RP | AR | *MYO7A* |
| 38 | 38-2 | 18 | F | Arab | Yes | Usher syndrome, sensorineural congenital hearing loss and RP | AR | *MYO7A* |
| 39 | 39-1 | 37 | M | Arab | Yes | Usher syndrome, sensorineural congenital hearing loss and RP | AR | *PCDH15* |
| 40 | 40-1 | 52 | M | Arab | Yes | Usher syndrome, sensorineural congenital hearing loss and RP | AR | *USH2A* |
| 41 | 41-1 | 59 | M | Arab | Yes | Usher syndrome, sensorineural congenital hearing loss and RP | AR | *USH2A* |
| 42 | 42-1 | 33 | M | Pakistani | Yes | Usher syndrome, sensorineural congenital hearing loss and RP | AR | *ADGRV1* |
| 43 | 43-1 | 27 | M | Arab | Yes | Bardet-Biedl syndrome | AR | *BBS2* |
| 43 | 43-2 | 21 | F | Arab | Yes | Bardet-Biedl syndrome | AR | *BBS2* |
| 44 | 44-1 | 17 | M | Arab | Yes | Bardet-Biedl syndrome | AR | *MKKS* |
| 45 | 45-1 | 17 | M | Arab | Yes | Bardet-Biedl syndrome, nystagmus, RP, polydactyly, obesity | AR | *BBS7* |
| 46 | 46-1 | 2 | M | Indian | No | Aniridia | AD | *PAX6* |
| 47 | 47-1 | 13 | F | Pakistani | Yes | Achromatopsia, hypermetropia | AR | *CNGB3* |
| 48 | 48-1 | 12 | M | Arab | Yes | Achromatopsia, bilateral myopic astigmatism | AR | *CNGB3* |
| 48 | 48-2 | 14 | M | Arab | Yes | Achromatopsia, bilateral astigmatism | AR | *CNGB3* |
| 49 | 49-1 | 17 | M | Arab | Yes | Achromatopsia, myopia | AR | *PDE6C* |
| 50 | 50-1 | 3 | M | Eqyptian | No | Oculocutaneous albinism, nystagmus, mild astigmatism | AD | *OCA2* |
| 51 | 51-1 | 39 | M | Arab | Yes | Oculocutaneous albinism, nystagmus, myopia | AR | *SLC24A5* |
| 52 | 52-1 | 8 | M | Egyptian | No | Retinoschisis | X-l | *RS1* |
| 53 | 53-1 | 16 | M | Arab | Yes | Retinoschisis | X-l | *RS1* |
| 54 | 54-1 | 34 | M | Arab | Yes | RP, myopia | AR | *NPHP4*^†^ |
| 55 | 55-1 | 37 | F | Arab | Yes | RCD | AR | *MERTK*^†^ |
| 56 | 56-1 | 9 | M | Arab | No | EORD, nystagmus | AR | *KIZ*^†^ |
|  |  |  |  |  |  |  | AR | *CNGA3*^†^ |
|  |  |  |  |  |  |  | AD | *RHO*^†^ |
| 57 | 57-1 | 25 | M | Pakistani | Yes | LCA | AR | *RPGRIP1*^‡^ |
| 58 | 58-1 | 26 | F | Arab | Yes | LCA | AR | *RPGRIP1*^‡^ |
| 58 | 58-2 | 28 | M | Arab | Yes | LCA | AR | *RPGRIP1*^‡^ |
| 59 | 59-1 | 18 | F | Japanese | No | Stargardt disease | AR | *ABCA4*^‡^ |
| 60 | 60-1 | 37 | F | Pakistani | Yes | Stargardt disease | AR | *ABCA4*^‡^ |
| 61 | 61-1 | 54 | F | Arab | Yes | RP | AR | *MERTK*^‡^ |
| 62 | 62-1 | 7 | F | Arab | Yes | Senior-Løken syndrome 5 | AR | *IQCB1*^‡^ |
| 63 | 63-1 | 42 | M | Arab | Yes | Retinoschisis | XL | *RS1*^‡^ |
| 64 | 64-1 | 11 | M | Pakistani | Yes | EORD, muscle contractures of the elbows |  | NPF |
| 65 | 65-1 | 60 | M | Indian | No | RP, moderate nuclear cataract |  | NPF |
| 66 | 66-1 | 24 | F | Sudanese | No | RP |  | NPF |
| 67 | 67-1 | 60 | M | Arab | Yes | Retinal dystrophy and optic atrophy |  | NPF |
| 68 | 68-1 | 12 | F | Indian | No | Fleck retinopathy, myopia |  | NPF |
| 69 | 69-1 | 43 | M | Arab | Yes | Pathological myopia with large areas of chorioretinal atrophy |  | NPF |
| 69 | 69-2 | 80 | F | Arab | Yes | Pathological myopia, bullous keratopathy right eye: macular degeneration |  | NPF |
| 70 | 70-1 | 18 | M | Arab | Yes | RCD |  | Did not proceed with testing |
| 71 | 71-1 | 15 | F | Arab | Yes | Stargardt disease |  | Did not proceed with testing |
| 71 | 71-2 | 20 | M | Arab | Yes | Stargardt disease |  | Did not proceed with testing |
| 72 | 72-1 | 37 | M | Arab | Yes | Stargardt disease |  | Did not proceed with testing |
| 73 | 73-1 | 42 | F | Arab | Yes | Macular dystrophy |  | Did not proceed with testing |
| 74 | 74-1 | 20 | M | Arab | Yes | Retinoschisis |  | Did not proceed with testing |
