# Supplementary material for: The genetic landscape of inherited eye disorders in 74 consecutive families from the United Arab Emirates
Source: Am J Med Genet C Semin Med Genet. 2020 Aug 11;184(3):762–72. doi: 10.1002/ajmg.c.31824 (PMC8432150; doi:10.1002/ajmg.c.31824)
Supplement: Supplementary file 2 — TABLE S2 Each variant identified in this cohort is listed below according to the American College of Medical Genetics and Genomics (ACMG) 2015 criteria (Richards et al., 2015). Fourteen novel variants in twelve genes, with three large duplications or deletions in three genes, are depicted in bold. †Variant(s) found but the diagnosis remains unconfirmed. [file AJMG-184-762-s001.docx]

**Supplementary table 2:** Each variant identified in this cohort is listed below according to the American College of Medical Genetics and Genomics (ACMG) 2015 criteria (Richards et al., 2015). Fourteen novel variants in twelve genes, with three large duplications or deletions in three genes, are depicted in bold. ^†^Variant(s) found but the diagnosis remains unconfirmed.

| Gene | Variant | Classification | ACMG 2015 criteria | Reference |
| --- | --- | --- | --- | --- |
| *ABCA4* | c.1610G>A p.(Arg537His) | Likely pathogenic | PS4, PM2, PM5, PP3, BS2 | (Jaakson et al., 2003) |
| *ABCA4* | c.1622T>C p.(Leu541Pro); c.3113C>T p.(Ala1038Val) | Pathogenic | PS3, PS4, PM3, PP3 | (Rozet et al., 1998); (Allikmets, 1997) |
| *ABCA4* | c.1714C>T p.(Arg572*) | Pathogenic | PVS1, PS4, PM2 | (Stenirri et al., 2008) |
| *ABCA4* | c.2382+4A>G | Likely pathogenic | PM2, PM4, PP1, PP3 | Report in LOVD |
| *ABCA4* | c.2570T>C p.(Leu857Pro) | Likely pathogenic | PS4, PM2, PP3 | (Khan et al., 2019) |
| *ABCA4* | c.319C>T p.(Arg107*) | Pathogenic | PVS1, PP3, PS4, PM2 | (Burke et al., 2010) |
| *ABCA4* | c.3898C>T p.(Arg1300*) | Pathogenic | PVS1, PP3, PS4, PM2 | (Rivera et al., 2000) |
| *ABCA4* | c.4793C>A p.(Ala1598Asp) | Likely pathogenic | PS4, PM2, PP1, PP3 | (Maugeri et al., 2000) |
| ***ABCA4*** | **c.5137_5138delinsAG p.(Gln1713Arg)** | Likely pathogenic | PS1, PM2, PP3 | This study |
| *ABCA4* | c.5461-10T>C | Pathogenic | PS3, PS4, PP3 | (Rivera et al., 2000) |
| *ABCA4* | c.5512C>G p.(His1838Asp) | Likely pathogenic | PS4, PM2, PP3 | (Burke et al., 2012) |
| *ABCA4* | c.5714+5G>A | Pathogenic | PS3, PS4, PM3 | (F. Cremers, 1998) |
| *ABCA4* | c.5882G>A p.(Gly1961Glu) | Pathogenic | PS3, PS4, PP3, PP4, BS2 | (Allikmets, 1997) |
| *ABCA4* | c.6148G>C p.(Val2050Leu) | Likely pathogenic | PP3, PS4, PM2, BS2 | (Allikmets, 1997) |
| *ABCA4* | c.6380C>T p.(Ser2127Phe) | Likely pathogenic | PS4, PM2, PP3 | (Khan et al., 2020) |
| *ABCA4* | c.6729+5_6729+19del p.(Phe2161Cysfs*3) | Pathogenic | PVS1, PS3, PS4, PM2, PP3 | (Littink et al., 2010) |
| ***ABCA4*** | **c.6820T>A p.(*2274Argext*34)** | Pathogenic | PVS1, PM2, PP3 | This study |
| *ADGRV1* | c.12798T>A p.(Tyr4266*) | Pathogenic | PVS1, PS4, PM2, PP3 | (Carss et al., 2017) |
| ***AGBL5*** | **c.313_319del p.(Gly105Profs*24)** | Pathogenic | PVS1, PM2, PP3 | This study |
| *AIPL1* | c.404dup p.(Asp136Glyfs*22) | Pathogenic | PVS1, PM2, PP1, PP3 | (Bryant et al., 2018) |
| *AIPL1* | c.834G>A p.(Trp278*) | Pathogenic | PVS1, PS4, PM2, PP1, PP3 | (Sohocki et al., 2000) |
| *BBS2* | c.117G>A p.(Lys39=) | Likely pathogenic | PS4, PM4, PP1, PP3 | (Deveault et al., 2011) |
| *BBS7* | c.968A>G p.(His323Arg) | Likely pathogenic | PS4, PM2, PP3 | (Badano et al., 2003) |
| ***CDHR1*** | **Deletion of the first six coding exons** | Uncertain significance | PVS1, PP3 | This study |
| *CNGA1* | c.1035dup p.(Arg346Thrfs*7) | Pathogenic | PVS1, PM2, PP3 | Report in ClinVar |
| *CNGA3* | c.1705C>T p.(Arg569Cys) | Uncertain significance | PM2, PM5, PP3 | This study^†^ |
| *CNGA3* | c.967G>C p.(Ala323Pro) | Uncertain significance | PM2, PM5, PP3 | This study^†^ |
| ***CNGB1*** | **c.2977-2del** | Pathogenic | PVS1, PM2, PP3 | This study |
| ***CNGB1*** | **c.973C>T p.(Gln325*)** | Pathogenic | PVS1, PM2, PP3 | This study |
| *CNGB3* | c.1063C>T p.(Arg355*) | Pathogenic | PVS1, PM2, PP1, PP3 | (Pentao et al., 1992) |
| *CNGB3* | c.1148del p.(Thr383Ilefs*13) | Pathogenic | PVS1, PS4, PM2, PP1, PP3, BS2 | (Kohl, 2000; Sundin et al., 2000) |
| *CRB1* | c.2506C>A p.(Pro836Thr) | Likely pathogenic | PS4, PM2, PP3 | (Henderson et al., 2011) |
| ***CRB1*** | **c.2842+1delinsAA** | Pathogenic | PVS1, PM2, PP3 | This study |
| *GUCY2D* | c.3056A>C p.(His1019Pro) | Likely pathogenic | PS4, PM2, PP3 | (Perrault et al., 2000) |
| ***HESX1*** | **c.450C>G p.(Asp150Glu)** | Uncertain significance | PM2, PP3, PP4 | This study |
| ***IFT172*** | **c.1156C>T p.(Arg386Trp)** | Uncertain significance | PM2, PP1, PP3 | This study |
| ***KCNJ13*** | **c.431T>C p.(Leu144Pro)** | Uncertain significance | PM2, PP1, PP3 | This study |
| *KCNV2* | c.427G>T p.(Glu143*) | Pathogenic | PVS1, PS4, PM2, PP1, PP3 | (Wu et al., 2006) |
| *KIZ* | c.583C>T p.(Arg195*) | Pathogenic | PVS1, PM2, PP3 | This study^†^ |
| *MERTK* | c.2214del p.(Cys738Trpfs*32) | Pathogenic | PVS1, PS4, PM2, PP1, PP3 | (Tschernutter et al., 2006) |
| *MERTK* | c.721C>T p.(Gln241*) | Pathogenic | PVS1, PS4, PM2, PP1, PP3 | (Srilekha et al., 2015) |
| *MERTK* | c.845-18G>A | Uncertain significance | PM2, PP3, BS2 | This study^†^ |
| *MERTK* | Multi-exon deletion | Pathogenic | PVS1, PM4, PP3 | This study^†^ |
| *MKKS* | c.295T>C p.(Cys99Arg) | Likely pathogenic | PS4, PM2, PP3 | (Billingsley et al., 2010) |
| *MYO7A* | c.5392C>T p.(Gln1798*) | Pathogenic | PVS1, PS4, PM2, PP1, PP3 | (Janecke et al., 1999) |
| *NPHP4* | c.955A>G p.(Ser319Gly) | Uncertain significance | PM2, PP3 | This study^†^ |
| *NR2E3* | c.932G>A p.(Arg311Gln) | Likely pathogenic | PS4, PM2, PP3 | (Haider et al., 2000) |
| ***OCA2*** | **c.890+1G>A** | Pathogenic | PVS1, PM2, PP3 | This study |
| *PAX6* | c.107_114dup p.(Pro39Glyfs*18) | Pathogenic | PVS1, PS4, PM2, PP3 | (Cross et al., 2020) |
| ***PCARE*** | **c.3668+2T>C** | Likely pathogenic | PM2, PM4, PP3 | This study |
| *PCDH15* | Deletion of the first three coding exons | Pathogenic | PVS1, PM4, PP3 | This study |
| *PDE6C* | c.490T>C p.(Phe164Leu) | Uncertain significance | PM2, PP3 | Report in ClinVar |
| *PROM1* | c.1557C>G p.(Tyr519*) | Pathogenic | PVS1, PS4, PM2, PP3 | (Abu-Safieh et al., 2013) |
| *RDH12* | c.139G>A p.(Ala47Thr) | Likely pathogenic | PS4, PM2, PP1, PP3 | (Thompson et al., 2005) |
| *RHO* | c.70T>C p.(Phe24Leu) | Uncertain significance | PM2, PP3 | This study^†^ |
| *RP1* | c.1047G>A p.(Trp349*) | Pathogenic | PVS1, PS4, PM2, PP1, PP3 | (Koyanagi et al., 2019) |
| *RP1* | c.1462del p.(Glu488Lysfs*44) | Pathogenic | PVS1, PM2, PP3 | Report in ClinVar |
| *RP1* | c.2219C>G p.(Ser740*) | Pathogenic | PVS1, PP3, PS4, PM2 | (Koyanagi et al., 2019) |
| *RP1* | c.310T>C p.(Tyr104His) | Likely pathogenic | PS4, PM2, PP1, PP3 | (Hariri et al., 2018) |
| *RPE65* | c.1451-2A>C | Pathogenic | PVS1, PS4, PM2, PP3 | (Li et al., 2020) |
| *RPGRIP1* | c.1107del p.(Glu370Asnfs*5) | Pathogenic | PVS1, PS4, PM2, PP3 | (Abu-Safieh et al., 2013) |
| *RS1* | c.304C>T p.(Arg102Trp) | Likely pathogenic | PS4, PM2, PP3 | (Dodds et al., 2006) |
| *RS1* | c.305G>A p.(Arg102Gln) | Likely pathogenic | PS4, PM2, PM5, PP3 | (The Retinoschisis Consortium, 1998) |
| *SDCCAG8* | c.1444del p.(Thr482Leufs*12) | Pathogenic | PVS1, PS4, PM2, PP3 | (Otto et al., 2010) |
| *SLC24A5* | c.328G>C p.(Gly110Arg) | Uncertain significance | PM2, PP3 | (Yousaf et al., 2020) |
| ***TEAD1*** | **Multi-exon (1-8) duplication** | Pathogenic | PVS1, PP1, PP3 | This study |
| ***TTLL5*** | **Multi-exons (16-26) deletion** | Uncertain significance | PVS1, PP3 | This study |
| *USH2A* | c.486-1G>C | Pathogenic | PVS1, PS4, PM2, PM4, PP3 | (F. P. M. Cremers et al., 2007) |

Abu-Safieh, L., Alrashed, M., Anazi, S., Alkuraya, H., Khan, A. O., Al-Owain, M., Al-Zahrani, J., Al-Abdi, L., Hashem, M., Al-Tarimi, S., Sebai, M.-A., Shamia, A., Ray-zack, M. D., Nassan, M., Al-Hassnan, Z. N., Rahbeeni, Z., Waheeb, S., Alkharashi, A., Abboud, E., … Alkuraya, F. S. (2013). Autozygome-guided exome sequencing in retinal dystrophy patients reveals pathogenetic mutations and novel candidate disease genes. *Genome Research*, *23*(2), 236–247. https://doi.org/10.1101/gr.144105.112

Allikmets, R. (1997). Mutation of the Stargardt Disease Gene (ABCR) in Age-Related Macular Degeneration. *Science*, *277*(5333), 1805–1807. https://doi.org/10.1126/science.277.5333.1805

Badano, J. L., Ansley, S. J., Leitch, C. C., Lewis, R. A., Lupski, J. R., & Katsanis, N. (2003). Identification of a Novel Bardet-Biedl Syndrome Protein, BBS7, That Shares Structural Features with BBS1 and BBS2. *The American Journal of Human Genetics*, *72*(3), 650–658. https://doi.org/10.1086/368204

Billingsley, G., Bin, J., Fieggen, K. J., Duncan, J. L., Gerth, C., Ogata, K., Wodak, S. S., Traboulsi, E. I., Fishman, G. A., Paterson, A., Chitayat, D., Knueppel, T., Millan, J. M., Mitchell, G. A., Deveault, C., & Heon, E. (2010). Mutations in chaperonin-like BBS genes are a major contributor to disease development in a multiethnic Bardet-Biedl syndrome patient population. *Journal of Medical Genetics*, *47*(7), 453–463. https://doi.org/10.1136/jmg.2009.073205

Bryant, L., Lozynska, O., Maguire, A. M., Aleman, T. S., & Bennett, J. (2018). Prescreening whole exome sequencing results from patients with retinal degeneration for variants in genes associated with retinal degeneration. *Clinical Ophthalmology (Auckland, N.Z.)*, *12*, 49–63. https://doi.org/10.2147/OPTH.S147684

Burke, T. R., Allikmets, R., Smith, R. T., Gouras, P., & Tsang, S. H. (2010). Loss of peripapillary sparing in non-group I Stargardt disease. *Experimental Eye Research*, *91*(5), 592–600. https://doi.org/10.1016/j.exer.2010.07.018

Burke, T. R., Fishman, G. A., Zernant, J., Schubert, C., Tsang, S. H., Smith, R. T., Ayyagari, R., Koenekoop, R. K., Umfress, A., Ciccarelli, M. L., Baldi, A., Iannaccone, A., Cremers, F. P. M., Klaver, C. C. W., & Allikmets, R. (2012). Retinal Phenotypes in Patients Homozygous for the G1961E Mutation in the *ABCA4* Gene. *Investigative Opthalmology & Visual Science*, *53*(8), 4458. https://doi.org/10.1167/iovs.11-9166

Carss, K. J., Arno, G., Erwood, M., Stephens, J., Sanchis-Juan, A., Hull, S., Megy, K., Grozeva, D., Dewhurst, E., Malka, S., Plagnol, V., Penkett, C., Stirrups, K., Rizzo, R., Wright, G., Josifova, D., Bitner-Glindzicz, M., Scott, R. H., Clement, E., … Yu, P. (2017). Comprehensive Rare Variant Analysis via Whole-Genome Sequencing to Determine the Molecular Pathology of Inherited Retinal Disease. *The American Journal of Human Genetics*, *100*(1), 75–90. https://doi.org/10.1016/j.ajhg.2016.12.003

Cremers, F. (1998). Autosomal recessive retinitis pigmentosa and cone-rod dystrophy caused by splice site mutations in the Stargardt’s disease gene ABCR. *Human Molecular Genetics*, *7*(3), 355–362. https://doi.org/10.1093/hmg/7.3.355

Cremers, F. P. M., Kimberling, W. J., Külm, M., de Brouwer, A. P., van Wijk, E., te Brinke, H., Cremers, C. W. R. J., Hoefsloot, L. H., Banfi, S., Simonelli, F., Fleischhauer, J. C., Berger, W., Kelley, P. M., Haralambous, E., Bitner-Glindzicz, M., Webster, A. R., Saihan, Z., De Baere, E., Leroy, B. P., … Kremer, H. (2007). Development of a genotyping microarray for Usher syndrome. *Journal of Medical Genetics*, *44*(2), 153–160. https://doi.org/10.1136/jmg.2006.044784

Cross, E., Duncan-Flavell, P. J., Howarth, R. J., Crooks, R. O., Thomas, N. S., & Bunyan, D. J. (2020). Screening of a large PAX6 cohort identified many novel variants and emphasises the importance of the paired and homeobox domains. *European Journal of Medical Genetics*, 103940. https://doi.org/10.1016/j.ejmg.2020.103940

Deveault, C., Billingsley, G., Duncan, J. L., Bin, J., Theal, R., Vincent, A., Fieggen, K. J., Gerth, C., Noordeh, N., Traboulsi, E. I., Fishman, G. A., Chitayat, D., Knueppel, T., Millán, J. M., Munier, F. L., Kennedy, D., Jacobson, S. G., Innes, A. M., Mitchell, G. A., … Héon, E. (2011). BBS genotype-phenotype assessment of a multiethnic patient cohort calls for a revision of the disease definition. *Human Mutation*, *32*(6), 610–619. https://doi.org/10.1002/humu.21480

Dodds, J. A., Srivastava, A. K., & Holden, K. R. (2006). Unusual phenotypic expression of an XLRS1 mutation in X-linked juvenile retinoschisis. *Journal of Child Neurology*, *21*(4), 331–333. https://doi.org/10.1177/08830738060210041901

Haider, N. B., Jacobson, S. G., Cideciyan, A. V., Swiderski, R., Streb, L. M., Searby, C., Beck, G., Hockey, R., Hanna, D. B., Gorman, S., Duhl, D., Carmi, R., Bennett, J., Weleber, R. G., Fishman, G. A., Wright, A. F., Stone, E. M., & Sheffield, V. C. (2000). Mutation of a nuclear receptor gene, NR2E3, causes enhanced S cone syndrome, a disorder of retinal cell fate. *Nature Genetics*, *24*(2), 127–131. https://doi.org/10.1038/72777

Hariri, A. H., Gui, W., Datoo O’Keefe, G. A., Ip, M. S., Sadda, S. R., & Gorin, M. B. (2018). Ultra-Widefield Fundus Autofluorescence Imaging of Patients with Retinitis Pigmentosa. *Ophthalmology Retina*, *2*(7), 735–745. https://doi.org/10.1016/j.oret.2017.10.018

Henderson, R. H., Mackay, D. S., Li, Z., Moradi, P., Sergouniotis, P., Russell-Eggitt, I., Thompson, D. A., Robson, A. G., Holder, G. E., Webster, A. R., & Moore, A. T. (2011). Phenotypic variability in patients with retinal dystrophies due to mutations in CRB1. *The British Journal of Ophthalmology*, *95*(6), 811–817. https://doi.org/10.1136/bjo.2010.186882

Jaakson, K., Zernant, J., Külm, M., Hutchinson, A., Tonisson, N., Glavač, D., Ravnik-Glavač, M., Hawlina, M., Meltzer, M. R., Caruso, R. C., Testa, F., Maugeri, A., Hoyng, C. B., Gouras, P., Simonelli, F., Lewis, R. A., Lupski, J. R., Cremers, F. P. M., & Allikmets, R. (2003). Genotyping microarray (gene chip) for the ABCR (ABCA4) gene. *Human Mutation*, *22*(5), 395–403. https://doi.org/10.1002/humu.10263

Janecke, A. R., Meins, M., Sadeghi, M., Grundmann, K., Apfelstedt-Sylla, E., Zrenner, E., Rosenberg, T., & Gal, A. (1999). Twelve novel myosin VIIA mutations in 34 patients with Usher syndrome type I: Confirmation of genetic heterogeneity. *Human Mutation*, *13*(2), 133–140. https://doi.org/10.1002/(SICI)1098-1004(1999)13:2<133::AID-HUMU5>3.0.CO;2-U

Khan, M., Cornelis, S. S., Khan, M. I., Elmelik, D., Manders, E., Bakker, S., Derks, R., Neveling, K., van de Vorst, M., Gilissen, C., Meunier, I., Defoort, S., Puech, B., Devos, A., Schulz, H. L., Stöhr, H., Grassmann, F., Weber, B. H. F., Dhaenens, C.-M., & Cremers, F. P. M. (2019). Cost-effective molecular inversion probe-based ABCA4 sequencing reveals deep-intronic variants in Stargardt disease. *Human Mutation*, *40*(10), 1749–1759. https://doi.org/10.1002/humu.23787

Khan, M., Cornelis, S. S., Pozo-Valero, M. D., Whelan, L., Runhart, E. H., Mishra, K., Bults, F., AlSwaiti, Y., AlTalbishi, A., De Baere, E., Banfi, S., Banin, E., Bauwens, M., Ben-Yosef, T., Boon, C. J. F., van den Born, L. I., Defoort, S., Devos, A., Dockery, A., … Cremers, F. P. M. (2020). Resolving the dark matter of ABCA4 for 1054 Stargardt disease probands through integrated genomics and transcriptomics. *Genetics in Medicine*. https://doi.org/10.1038/s41436-020-0787-4

Kohl, S. (2000). Mutations in the CNGB3 gene encoding the beta-subunit of the cone photoreceptor cGMP-gated channel are responsible for achromatopsia (ACHM3) linked to chromosome 8q21. *Human Molecular Genetics*, *9*(14), 2107–2116. https://doi.org/10.1093/hmg/9.14.2107

Koyanagi, Y., Akiyama, M., Nishiguchi, K. M., Momozawa, Y., Kamatani, Y., Takata, S., Inai, C., Iwasaki, Y., Kumano, M., Murakami, Y., Omodaka, K., Abe, T., Komori, S., Gao, D., Hirakata, T., Kurata, K., Hosono, K., Ueno, S., Hotta, Y., … Sonoda, K.-H. (2019). Genetic characteristics of retinitis pigmentosa in 1204 Japanese patients. *Journal of Medical Genetics*, *56*(10), 662–670. https://doi.org/10.1136/jmedgenet-2018-105691

Li, S., Xiao, X., Yi, Z., Sun, W., Wang, P., & Zhang, Q. (2020). RPE65 mutation frequency and phenotypic variation according to exome sequencing in a tertiary centre for genetic eye diseases in China. *Acta Ophthalmologica*, *98*(2). https://doi.org/10.1111/aos.14181

Littink, K. W., Koenekoop, R. K., van den Born, L. I., Collin, R. W. J., Moruz, L., Veltman, J. A., Roosing, S., Zonneveld, M. N., Omar, A., Darvish, M., Lopez, I., Kroes, H. Y., van Genderen, M. M., Hoyng, C. B., Rohrschneider, K., van Schooneveld, M. J., Cremers, F. P. M., & den Hollander, A. I. (2010). Homozygosity mapping in patients with cone-rod dystrophy: Novel mutations and clinical characterizations. *Investigative Ophthalmology & Visual Science*, *51*(11), 5943–5951. https://doi.org/10.1167/iovs.10-5797

Maugeri, A., Klevering, B. J., Rohrschneider, K., Blankenagel, A., Brunner, H. G., Deutman, A. F., Hoyng, C. B., & Cremers, F. P. M. (2000). Mutations in the ABCA4 (ABCR) Gene Are the Major Cause of Autosomal Recessive Cone-Rod Dystrophy. *The American Journal of Human Genetics*, *67*(4), 960–966. https://doi.org/10.1086/303079

Otto, E. A., Hurd, T. W., Airik, R., Chaki, M., Zhou, W., Stoetzel, C., Patil, S. B., Levy, S., Ghosh, A. K., Murga-Zamalloa, C. A., van Reeuwijk, J., Letteboer, S. J. F., Sang, L., Giles, R. H., Liu, Q., Coene, K. L. M., Estrada-Cuzcano, A., Collin, R. W. J., McLaughlin, H. M., … Hildebrandt, F. (2010). Candidate exome capture identifies mutation of SDCCAG8 as the cause of a retinal-renal ciliopathy. *Nature Genetics*, *42*(10), 840–850. https://doi.org/10.1038/ng.662

Pentao, L., Lewis, R. A., Ledbetter, D. H., Patel, P. I., & Lupski, J. R. (1992). Maternal uniparental isodisomy of chromosome 14: Association with autosomal recessive rod monochromacy. *American Journal of Human Genetics*, *50*(4), 690–699.

Perrault, I., Rozet, J.-M., Gerber, S., Ghazi, I., Ducroq, D., Souied, E., Leowski, C., Bonnemaison, M., Dufier, J.-L., Munnich, A., & Kaplan, J. (2000). Spectrum of retGC1 mutations in Leber’s congenital amaurosis. *European Journal of Human Genetics*, *8*(8), 578–582. https://doi.org/10.1038/sj.ejhg.5200503

Richards, S., Bale, S., Bick, D., Das, S., Gastier-Foster, J., Grody, W. W., Hegde, M., Lyon, E., Spector, E., Voelkerding, K., Rehm, H. L., & ; on behalf of the ACMG Laboratory Quality Assurance Committee. (2015). Standards and guidelines for the interpretation of sequence variants: A joint consensus recommendation of the American College of Medical Genetics and Genomics and the Association for Molecular Pathology. *Genetics in Medicine*, *17*(5), 405–423. https://doi.org/10.1038/gim.2015.30

Rivera, A., White, K., Stöhr, H., Steiner, K., Hemmrich, N., Grimm, T., Jurklies, B., Lorenz, B., Scholl, H. P., Apfelstedt-Sylla, E., & Weber, B. H. (2000). A comprehensive survey of sequence variation in the ABCA4 (ABCR) gene in Stargardt disease and age-related macular degeneration. *American Journal of Human Genetics*, *67*(4), 800–813. https://doi.org/10.1086/303090

Rozet, J.-M., Gerber, S., Souied, E., Perrault, I., Châtelin, S., Ghazi, I., Leowski, C., Dufier, J.-L., Munnich, A., & Kaplan, J. (1998). Spectrum of ABCR gene mutations in autosomal recessive macular dystrophies. *European Journal of Human Genetics*, *6*(3), 291–295. https://doi.org/10.1038/sj.ejhg.5200221

Sohocki, M. M., Bowne, S. J., Sullivan, L. S., Blackshaw, S., Cepko, C. L., Payne, A. M., Bhattacharya, S. S., Khaliq, S., Qasim Mehdi, S., Birch, D. G., Harrison, W. R., Elder, F. F. B., Heckenlively, J. R., & Daiger, S. P. (2000). Mutations in a new photoreceptor-pineal gene on 17p cause Leber congenital amaurosis. *Nature Genetics*, *24*(1), 79–83. https://doi.org/10.1038/71732

Srilekha, S., Arokiasamy, T., Srikrupa, N. N., Umashankar, V., Meenakshi, S., Sen, P., Kapur, S., & Soumittra, N. (2015). Homozygosity Mapping in Leber Congenital Amaurosis and Autosomal Recessive Retinitis Pigmentosa in South Indian Families. *PLOS ONE*, *10*(7), e0131679. https://doi.org/10.1371/journal.pone.0131679

Stenirri, S., Alaimo, G., Manitto, M. P., Brancato, R., Ferrari, M., & Cremonesi, L. (2008). Are microarrays useful in the screening of ABCA4 mutations in Italian patients affected by macular degenerations? *Clinical Chemistry and Laboratory Medicine*, *46*(9). https://doi.org/10.1515/CCLM.2008.248

Sundin, O. H., Yang, J.-M., Li, Y., Zhu, D., Hurd, J. N., Mitchell, T. N., Silva, E. D., & Maumenee, I. H. (2000). Genetic basis of total colourblindness among the Pingelapese islanders. *Nature Genetics*, *25*(3), 289–293. https://doi.org/10.1038/77162

The Retinoschisis Consortium. (1998). Functional Implications of the Spectrum of Mutations Found in 234 Cases With X-linked Juvenile Retinoschisis (XLRS). *Human Molecular Genetics*, *7*(7), 1185–1192. https://doi.org/10.1093/hmg/7.7.1185

Thompson, D. A., Janecke, A. R., Lange, J., Feathers, K. L., Hübner, C. A., McHenry, C. L., Stockton, D. W., Rammesmayer, G., Lupski, J. R., Antinolo, G., Ayuso, C., Baiget, M., Gouras, P., Heckenlively, J. R., den Hollander, A., Jacobson, S. G., Lewis, R. A., Sieving, P. A., Wissinger, B., … Gal, A. (2005). Retinal degeneration associated with RDH12 mutations results from decreased 11- cis retinal synthesis due to disruption of the visual cycle. *Human Molecular Genetics*, *14*(24), 3865–3875. https://doi.org/10.1093/hmg/ddi411

Tschernutter, M., Jenkins, S. A., Waseem, N. H., Saihan, Z., Holder, G. E., Bird, A. C., Bhattacharya, S. S., Ali, R. R., & Webster, A. R. (2006). Clinical characterisation of a family with retinal dystrophy caused by mutation in the Mertk gene. *The British Journal of Ophthalmology*, *90*(6), 718–723. https://doi.org/10.1136/bjo.2005.084897

Wu, H., Cowing, J. A., Michaelides, M., Wilkie, S. E., Jeffery, G., Jenkins, S. A., Mester, V., Bird, A. C., Robson, A. G., Holder, G. E., Moore, A. T., Hunt, D. M., & Webster, A. R. (2006). Mutations in the Gene KCNV2 Encoding a Voltage-Gated Potassium Channel Subunit Cause “Cone Dystrophy with Supernormal Rod Electroretinogram” in Humans. *The American Journal of Human Genetics*, *79*(3), 574–579. https://doi.org/10.1086/507568

Yousaf, S., Sethna, S., Chaudhary, M. A., Shaikh, R. S., Riazuddin, S., & Ahmed, Z. M. (2020). Molecular characterization of *SLC24A5* variants and evaluation of Nitisinone treatment efficacy in a zebrafish model of OCA6. *Pigment Cell & Melanoma Research*, pcmr.12879. https://doi.org/10.1111/pcmr.12879
